# Radiosynthesis and Evaluation of ^18^F‑Labeled Deuterated Radioligand for Positron Emission Tomography Imaging of Cholesterol 24-Hydroxylase

**DOI:** 10.1021/acsmedchemlett.5c00740

**Published:** 2026-01-20

**Authors:** Yinlong Li, Zhendong Song, Haofeng Shi, Taoqian Zhao, Jiahui Chen, Xin Zhou, Qilong Hu, Xiaoyan Li, Lingxin Meng, Ruihu Song, Zhenkun Sun, Chongjiao Li, Achi Haider, Hongjie Yuan, Steven H. Liang

**Affiliations:** ∇ Department of Radiology and Imaging Sciences, 1371 Emory University, 1364 Clifton Road, Atlanta, Georgia 30322, United States; ‡ Department of Pharmacology and Chemical Biology, Emory University School of Medicine, Atlanta, Georgia 30322, United States; § Wallace H. Coulter Department of Biomedical Engineering, Georgia Institute of Technology and Emory University, Atlanta, Georgia 30332, United States

**Keywords:** Cholesterol homeostasis, Cholesterol 24-hydroxylase, Fluorine-18, Positron emission tomography, Molecular imaging, Neurodegeneration

## Abstract

Brain cholesterol homeostasis is critical for neuronal function and primarily regulated by cholesterol 24-hydroxylase (CYP46A1). Dysregulation of CYP46A1 has been implicated in Alzheimer’s disease (AD) and Huntington’s disease (HD). Building on the clinically validated positron emission tomography (PET) tracer [^18^F]­CHL-2205, we designed a deuterated isotopologue, CHL-2205*-d*_3_, targeting the amide *N*-methyl group to enhance stability and enable mechanistic studies. Compound **5** exhibited high CYP46A1 affinity (IC_50_ = 0.38 nM; *K*_i_ = 0.22 nM). Radiosynthesis via copper-mediated [^18^F]­fluorination afforded [^18^F]**5** in 31.5 ± 1.5% non-decay-corrected radiochemical yield and high molar activity (>95 GBq/μmol). Autoradiography and PET imaging in mice demonstrated robust brain uptake, heterogeneous regional distribution, and specific target engagement. Radiometabolite analysis confirmed that brain radioactivity was mainly attributable to intact [^18^F]**5**, with a pharmacokinetics comparable to that of [^18^F]­CHL-2205. [^18^F]**5** preserves [^18^F]­CHL-2205 imaging performance and provides a deuterated PET tool for quantitative bioanalysis and integrated PET–deuterium metabolic imaging (DMI) studies of brain cholesterol metabolism.

## Graphical abstract

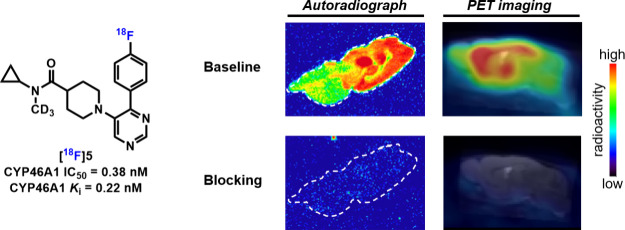

Cholesterol is an essential structural component of cell membranes and is widely distributed across tissues throughout the human body.^1,2^ Notably, the brain contains the highest concentration of cholesterol, accounting for approximately 23% of the total body pool.^3^ However, the restrictive nature of the blood–brain barrier (BBB) prevents cholesterol exchange between the plasma and the central nervous system (CNS).^4,5^ As a result, brain cholesterol is synthesized *in situ*, and its clearance depends on tightly regulated CNS-specific metabolic pathways.^6^ The primary metabolic pathway cholesterol in the brain involve the enzymes CYP11A1, CYP27A1, CYP7A1, and CYP46A1.^7^ Among them, CYP46A1 (cholesterol 24-hydroxylase or CH24H), predominantly distributed in the cerebral cortex, hippocampus, and striatum,^8^ is considered the key cholesterol-metabolizing enzyme in the CNS.^9^ CYP46A1 catalyzes the conversion of cholesterol into 24-hydroxycholesterol (24-OHC),^10^ which can efficiently cross the BBB to enter systemic circulation and is subsequently metabolized in the liver.^11^ This mechanism plays a crucial role in facilitating cholesterol elimination from the brain and maintaining cholesterol homeostasis.^12^ Dysregulation of CYP46A1 has been closely linked to various neurological diseases.^13^ In Alzheimer’s disease (AD), physiological levels of 24-OHC (1–10 μM) activate the liver X receptor (LXR), thereby reducing amyloid-β (Aβ) production and tau hyperphosphorylation while promoting neuroprotective responses.^14,15^ Pharmacological or genetic enhancement of CYP46A1 activity increases 24-OHC production, mitigates pathology, and improves cognitive function.^16,17^ Moreover, CYP46A1 upregulation has demonstrated neuroprotective benefits in Huntington’s disease (HD) models.^18^ Therefore, CYP46A1 has emerged as a promising therapeutic target and holds great significance in drug discovery and neurodegenerative disease therapy.^19–21^

Positron emission tomography (PET) is a powerful molecular imaging technique that provides a noninvasive and highly sensitive approach for quantifying and visualizing physiological and pathological processes living subjects.^22–25^ The development of PET tracers targeting CYP46A1 has facilitated the assessment of its distribution and activity in the brain, offering valuable tools for early diagnosis and therapeutic evaluation in neurodegenerative diseases.^26^ To date, several PET tracers for CYP46A1 have been developed (Figure 1A). The first-generation ^11^C-labeled tracer [^11^C]**1** was synthesized via an ‘in-loop’ [^11^C]­CO_2_ fixation method. However, it exhibited relatively low binding affinity and limited brain uptake, rendering it unsuitable for clinical applications.^27^ Notable CYP46A1-targeted PET tracers include [^18^F]**3g** and [^18^F]­T-008, which were developed and exhibited high affinity and strong uptake in CYP46A1-rich brain regions.^28,29^ More recently, a structurally distinct ligand, [^18^F]­CHL2310, was successfully validated for specific binding and favorable kinetics in rodents and nonhuman Primates (NHPs).^30,31^ We have recently reported on the development of the ^18^F-labeled PET tracer, [^18^F]­Cholestify ([^18^F]­CHL-2205), which demonstrated high affinity and specificity for CYP46A1.^32^ PET imaging studies in NHPs and humans confirmed a distribution pattern consistent with CYP46A1 expression. Despite these favorable characteristics, [^18^F]­CHL-2205 still contains a classical metabolic soft spot in the form of its *N*-methyl amide. Oxidative *N*-demethylation at such sites is a common clearance pathway mediated by cytochrome P450 enzymes and can, in principle, contribute to reduced parent fraction in plasma and the formation of radiometabolites that complicate quantitative PET analysis across species. Selective replacement of metabolically labile C–H bonds by C–D is a well-established medicinal chemistry strategy to attenuate oxidative metabolism through the deuterium kinetic isotope effect, while leaving steric and electronic properties essentially unchanged.^33^ In CNS-targeted PET, metabolic processes occurring in the periphery can be consequential even when brain metabolic fraction is minimal, because they modulate the fraction of intact tracer available in blood and thereby the time-dependent delivery of parent radioligand to the CNS. Further, substantial peripheral metabolism complicates simplified kinetic modeling protocols that rely on an image-derived input function in the absence of arterial sampling. Consequently, improvements in systemic stability can translate into altered apparent brain uptake kinetics and pay the way for simplified quantification methods that do not require arterial sampling, providing an additional rationale to examine targeted deuteration for stability assessments. Guided by this rationale, we designed the *N*-[methyl-*d*_3_] analog **5** ([^18^F]­CHL-2205-*d*_3_) and developed its ^18^F-labeled version [^18^F]**5** (Figure 1B). In the present work, we show that **5** maintains subnanomolar affinity for CYP46A1, favorable physicochemical and ADME (ADME, Absorption, Distribution, Metabolism, Excretion) properties, and robust brain penetration. Across *in vitro* autoradiography, mouse PET imaging, whole-body biodistribution, and radiometabolite analyses, [^18^F]**5** exhibits a regional distribution and kinetic profile closely aligned with those previously reported for [^18^F]­CHL-2205, indicating that *N*-methyl deuteration does not perturb target engagement while delivering a radioligand with excellent brain metabolic stability. Indeed, [^18^F]**5** broadens the chemical toolbox for CYP46A1 imaging and provides an instructive case study on the impact of precision deuteration on a highly optimized CNS PET scaffold, thereby opening avenues for multimodality imaging that harnesses both PET and deuterium metabolic imaging (DMI) techniques.^34^

**Figure 1:**
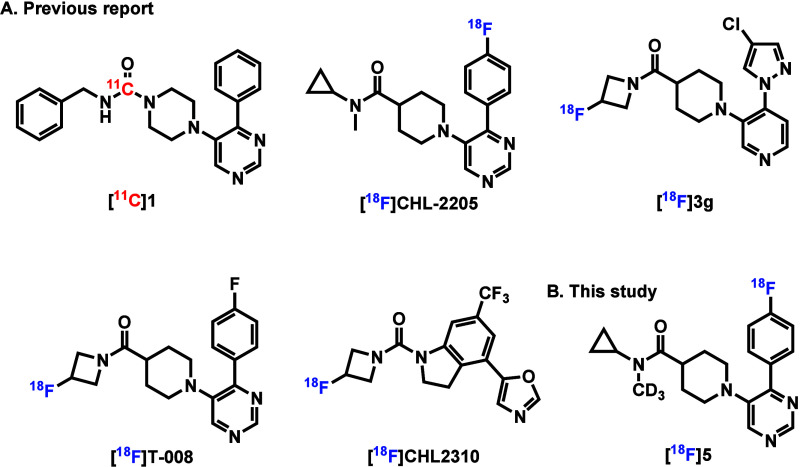
Representative CYP46A1 PET tracers.

## Results and Discussion

### Chemistry

The synthesis of compound **5** began with commercially available 1-(4-(4-fluorophenyl)­pyrimidin-5-yl)­piperidine-4-carboxylic acid (**2**), which was coupled with cyclopropylamine (**3**) to afford amide **4** in 88% yield. Subsequent deuterated methylation with NaH and CD_3_I produced the final compound **5** in 93% yield and >99% purity (Scheme 1).

**Scheme 1:**
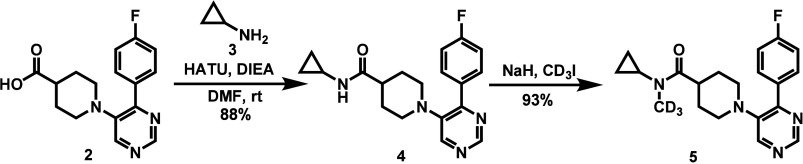
Synthesis of CYP46A1 Inhibitor **5**

### Pharmacology

The pharmacology and absorption, distribution, metabolism, and excretion (ADME) characteristics of compound **5** are presented in Table 1. To assess its binding affinity toward CYP46A1, a competitive radioligand binding assay was conducted on rat striatal tissue. As illustrated in Table 1, compound **5** displayed high inhibitory potency, with an IC_50_ of 0.38 nM and a *K*_i_ of 0.22 nM. In silico analyses further indicated favorable characteristics for brain penetration. Compound **5** displayed a molecular weight (MW) of 357.45 (<500), moderate lipophilicity (logP = 2.4), and TPSA (Topological Polar Surface Area) = 49.33 (<90). The experimentally measured logD = 2.82 (by the “shake-flask” method) also falls within the optimal range (2–3).^35^ The absence of hydrogen-bond donors (HBD = 0), brain/plasma partition coefficient (log BB = −0.19 > −1) and multiparameter optimization (MPO = 5.8 > 4.0) values meet reported thresholds, suggesting its CNS drug-like properties.^36,37^ In addition, compound **5** demonstrated excellent plasma stability, with half-lives (*t*_1/2_) of 155.6 min (human) and 110.4 min (rat), providing a rationale to further evaluate its stability across additional biological systems. The unbound brain-to-plasma ratio (*f*_u_ brain = 13.7 > 1) indicates good brain exposure. The MDCK-MDR1 cell permeability assay determined an efflux ratio (P_app_ B–A/P_app_ A–B) = 0.95 (<3.0), confirming that compound **5** is not a substrate of efflux transporters and is consistent with BBB permeability requirements.^38^ Furthermore, an *in vitro* off-target binding panel against 58 key CNS targets revealed no significant off-target interactions (Figure S1). Notably, the affinity and selectivity profile of **5** closely mirrors that of the nondeuterated CHL-2205 ligand (*K*_i_ = 0.26 nM),^32^ indicating that *N-*[methyl*-d*_3_] substitution does not measurably perturb the interaction with CYP46A1.

**Table 1:** Pharmacology and ADME Profiles of Compound **5**

**Parameters**	**Values**	**Parameters**	**Values**
IC_50_ (nM)	0.38	LogBB^b^	–0.19
*K*_i_ (nM)	0.22	MPO score^b^	5.8
MW^a^	357.45	hPlasma *t*_1/2_ (min)	155.6
logP^b^	2.40	rPlasma *t*_1/2_ (min)	110.4
logD^c^	2.82	(*f*_u_)% in rat brain	13.7
tPSA^b^	49.33	Papp(B-A)/Papp(A-B)	0.95
HBD^b^	0		

a Values were calculated with ChemDraw 21.0 software.

b Values were predictesd with ACD/laboratories.

c Determined by the “shake flask” method. hPlasma = human plasma; rPlasma = rat plasma.

### Radiochemistry

Considering the aryl-fluoro substituent in compound **5** that enables a fluorine-18 incorporation, we designed compound **13** as the radiolabeling precursor. The synthesis of precursor **13** was initiated from compound **6**, which first underwent coupling with amine **7** to afford intermediate **8** in 90% yield. An intermolecular cyclization with formimidamide subsequently provided compound **9** in 40% yield. Hydrolysis of the ester in **9** with 2N NaOH furnished the corresponding acid **10** in 90% yield, which was then coupled with amine **3** to obtain intermediate **11**. CD_3_-methylation of **11** produced compound **12** in 57% yield, and a Miyaura borylation reaction delivered precursor **13** in 42% yield (Scheme 2A). The radiosynthesis of [^18^F]**5** was carried out via a copper-mediated ^18^F-radiofluorination in *N*,*N*-dimethylacetamide (DMAC)/*n*-BuOH (v/v = 2:1) at 110 °C for 15 min (Scheme 2B). [^18^F]**5** was obtained in 31.5 ± 1.5% nondecay-corrected radiochemical yield with molar activity >95 GBq/μmol. Structural identity of [^18^F]**5** was verified by coinjection with the corresponding unlabeled reference compound **5** (Scheme 2C). Furthermore, we extended the *in vitro* studies using [^18^F]**5** to further assess its stability. As shown in Figure 2, [^18^F]**5** exhibited excellent stability in mouse, rat, NHP, and human serums, comparable to that of [^18^F]­CHL-2205. These findings demonstrate that [^18^F]**5** maintains consistent *in vitro* stability across multiple species, supporting its suitability for subsequent *in vivo* studies.

**Scheme 2:**
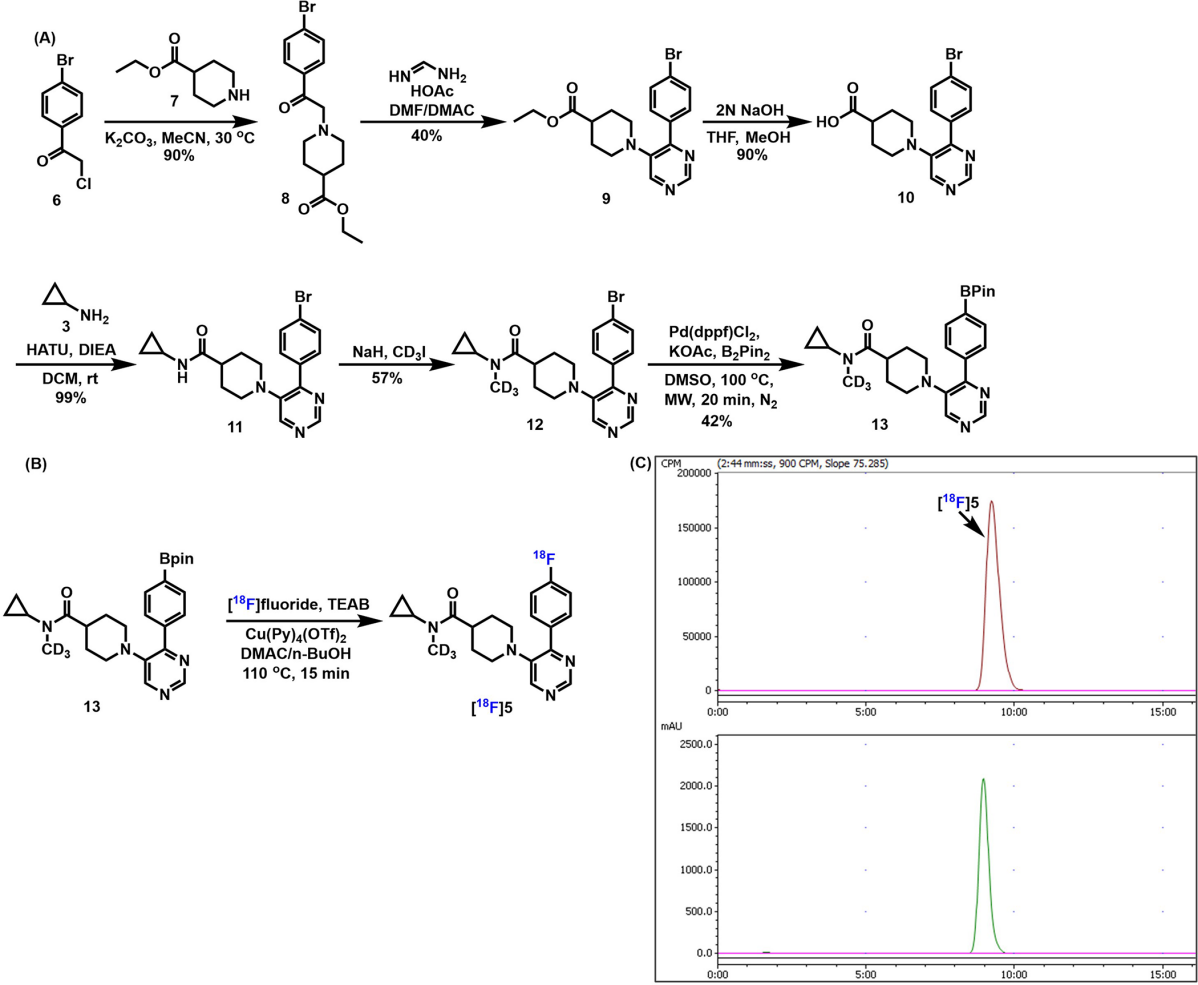
Radiochemistry. (A) Synthesis of precursor **13**. (B) Radiosynthesis of [^18^F]**5**. (C) Co-injection of [^18^F]**5** with the unlabeled reference compound **5**.

**Figure 2:**
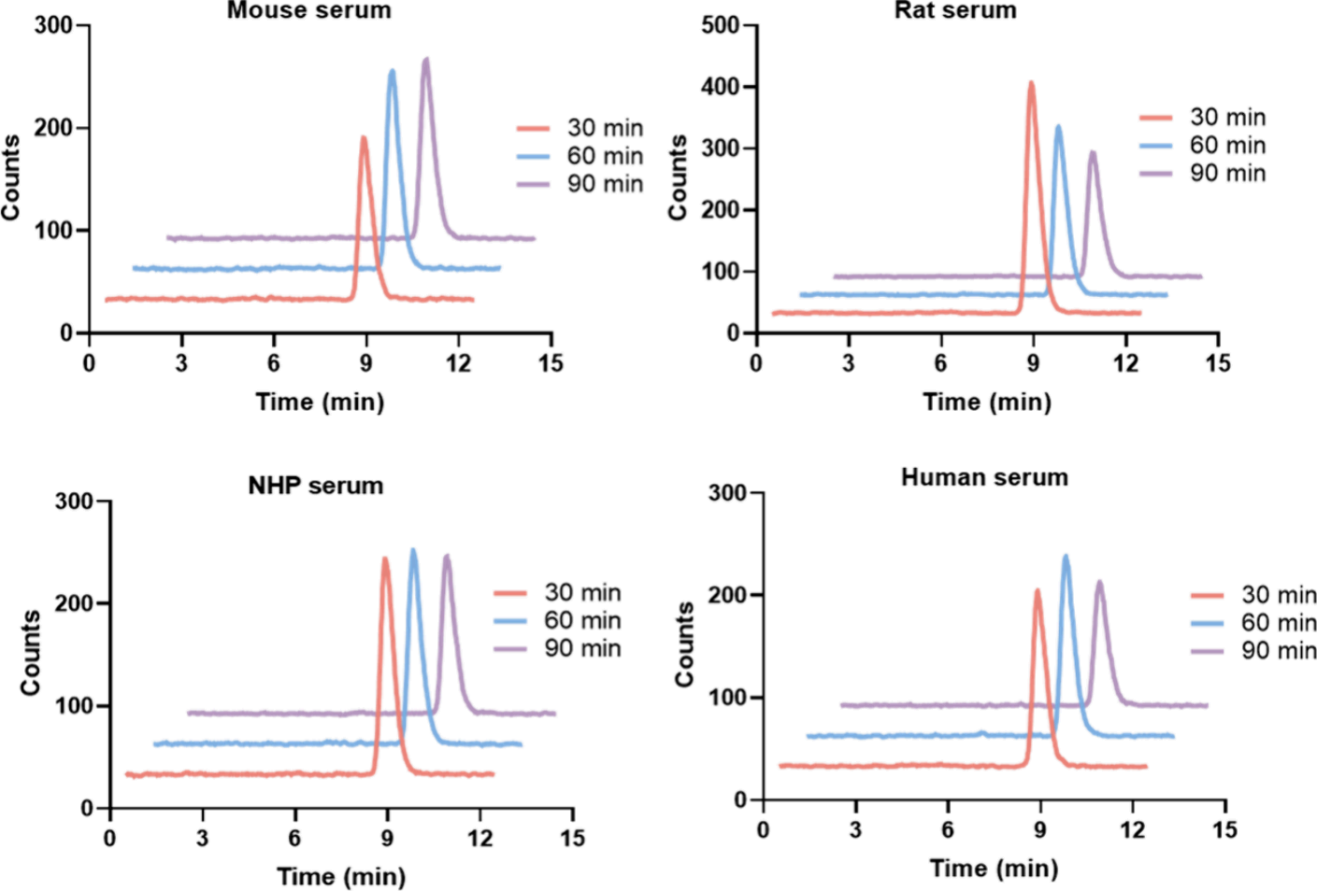
*In vitro* stability study of [^18^F]**5** in mouse, rat, NHP, and human serums.

### Radiometabolite Analysis

To further assess *in vivo* performance, *ex vivo* radiometabolite analysis of [^18^F]**5** was performed in the mouse brain and plasma 30 min postinjection and compared with [^18^F]­CHL-2205. As shown in Figure 3A&B, the intact parent [^18^F]**5** represented 93.6% of the total radioactivity in the brain and 13.3% in the plasma. These findings indicate that [^18^F]**5** exhibits excellent metabolic stability in the brain, and the radioactivity detected in brain primarily reflects the parent [^18^F]**5**, supporting reliable quantitative PET analysis. For comparison, the previously reported nondeuterated analog [^18^F]­CHL-2205 also exhibited negligible radiometabolite formation in mouse brain, with essentially all radioactivity attributable to intact parent tracer at similar time points (Figure 3C&D).^32^ Radiometabolite profiling was performed not only to confirm the high fraction of intact tracer in brain but also to assess whether peripheral metabolism could limit the availability of parent radioligand in blood. Such systemic processes can influence PET kinetics by shaping the input function and, thereby affecting the delivery of intact tracer to the CNS and precluding simplified kinetic modeling in the absence of arterial sampling. In a head-to-head comparison, however, [^18^F]­CHL-2205 and [^18^F]­CHL-2205-*d*_3_ exhibited comparable plasma and brain radiometabolite profiles, indicating that *N-*methyl deuteration does not meaningfully alter the systemic availability of intact tracer under the conditions tested. Instead, [^18^F]**5** preserves the favorable brain signal of [^18^F]­CHL-2205, while providing an isotopically labeled variant suitable for probing the role of precision deuteration in CYP46A1 PET imaging. It should be emphasized that the plasma profiles in Figure 3 were obtained *ex vivo* after *in vivo* tracer administration and therefore reflect a snapshot of circulating radiometabolites formed by whole-body metabolism – potentially in liver and other metabolically active organs – and subsequently released into the blood. This systemic biotransformation is not captured by the *in vitro* serum incubation assay (Figure 2), which primarily probes stability in the serum matrix under static conditions.

**Figure 3:**
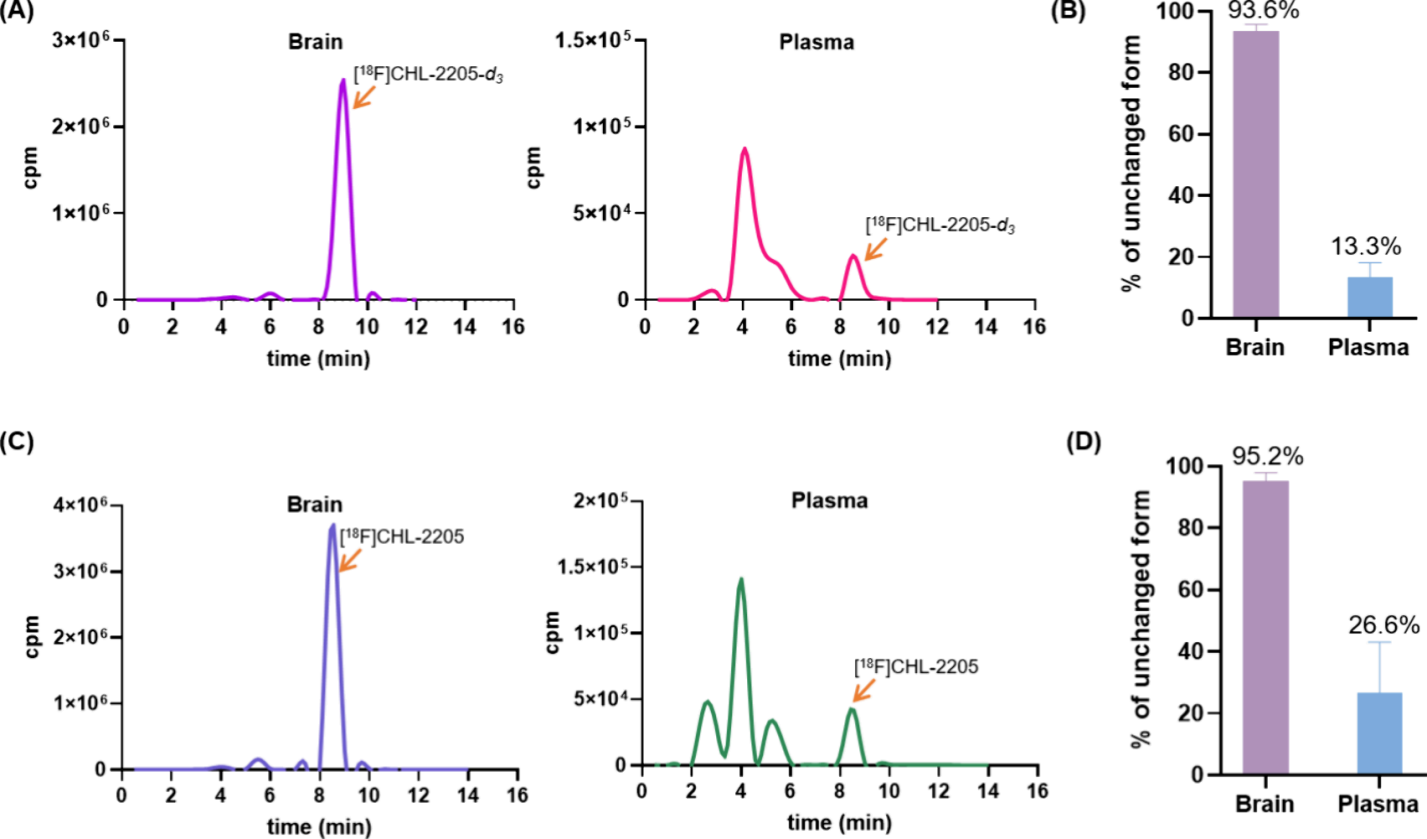
Representative radio-HPLC chromatograms of [^18^F]­CHL-2205-*d*_3_ (A) and [^18^F]­CHL-2205 (C) in CD-1 mouse brain and plasma at 30 min postinjection, and the corresponding percentages of unchanged tracer in brain and plasma (B, D).

From a design perspective, the present data suggest that *N-*demethylation is not a dominant clearance pathway for this chemotype in the mouse brain at PET microdosing, as targeted deuteration at the amide *N-*methyl does not yield a marked gain in apparent metabolic stability compared with [^18^F]­CHL-2205. This insight will be valuable when considering further modifications of CYP46A1 ligands, where emphasis can now be shifted toward other potential metabolic soft spots. The deuterated ligand also enables future multimodal studies on PET and MR systems via deuterium metabolic imaging, which uses deuterium-enriched substrates to generate three-dimensional maps of metabolic fluxes by magnetic resonance spectroscopic imaging.^39^ However, DMI is fundamentally distinct from PET in that it relies on MR detection of ^2^H-labeled substrates and metabolites and therefore typically requires substrate administration at concentrations orders of magnitude higher than PET microdosing. In principle, a hybrid PET/MR experiment could attempt to increase the deuterium mass by coadministering [^18^F]**5** together with additional nonradioactive deuterated CHL-2205-*d*_3_. Nonetheless, this approach would necessarily lower the effective molar activity and increase the injected mass dose, which for a high-affinity CNS radioligand could lead to partial CYP46A1 occupancy, reduced specific binding, and diminished PET signal-to-noise. Thus, while [^18^F]**5** provides an isotopically labeled analogue that may facilitate future methodological explorations, substantial challenges remain for simultaneous PET/DMI implementations, and dedicated dose-finding and safety studies would be required to establish feasibility and translational relevance.

### *In Vitro* Autoradiography Study

To evaluate the regional distribution and binding selectivity of [^18^F]**5**, we conducted *in vitro* autoradiography studies using rat brain tissue sections (Figure 4). The tracer exhibited a heterogeneous distribution, with high and regionally distinct radioactive accumulation in the thalamus, striatum, cortex, and hippocampus, while the cerebellum exhibited minimal signal. This distribution is closely matched with known CYP46A1 expression profiles.^40,41^ To further assess binding selectivity, coincubation with cold reference compound **5** or the validated CYP46A1 inhibitor Soticlestat significantly reduced radioactivity across all examined brain regions, indicating specific and saturable binding to CYP46A1. These results support the potential of [^18^F]**5** as a promising CYP46A1-targeted neuroimaging probe for further development.

**Figure 4:**
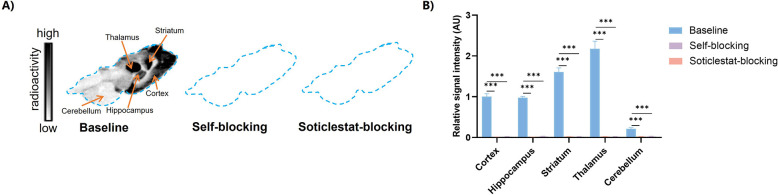
Autoradiography studies. (A) Representative *in vitro* autoradiograph of [^18^F]**5** under baseline and blocking conditions (blocker = 10 μM). (B) Quantitative analysis of *in vitro* autoradiograph with [^18^F]**5**.

### PET Imaging Study in Mice

In the next step, we evaluated the *in vivo* PET imaging performance of [^18^F]**5** in mice, and the results are summarized in Figure 5. Whole-brain baseline PET images summed over 0–60 min revealed that [^18^F]**5** readily crossed the BBB and exhibited a heterogeneous distribution pattern consistent with the autoradiography results (Figure 5A). The corresponding time–activity curves (TACs) demonstrated rapid brain uptake followed by gradual washout in CYP46A1-enriched regions, including the cortex, hippocampus, striatum, and thalamus (Figure 5B&C). To confirm target engagement, blocking studies were performed by pretreating mice with the reference compound **5** (1 mg/kg, self-blocking) or soticlestat (1 mg/kg). Both blocking conditions substantially decreased tracer uptake and led to a homogeneous regional distribution (Figure 5A&B). Quantification of the area under the curve (AUC_0–60 min_) further demonstrated that [^18^F]**5** uptake was reduced by approximately 82–89% in CYP46A1-rich regions (cortex, hippocampus, striatum, and thalamus) under both blocking conditions, indicating specific binding to CYP46A1 (Figure 5D).

**Figure 5:**
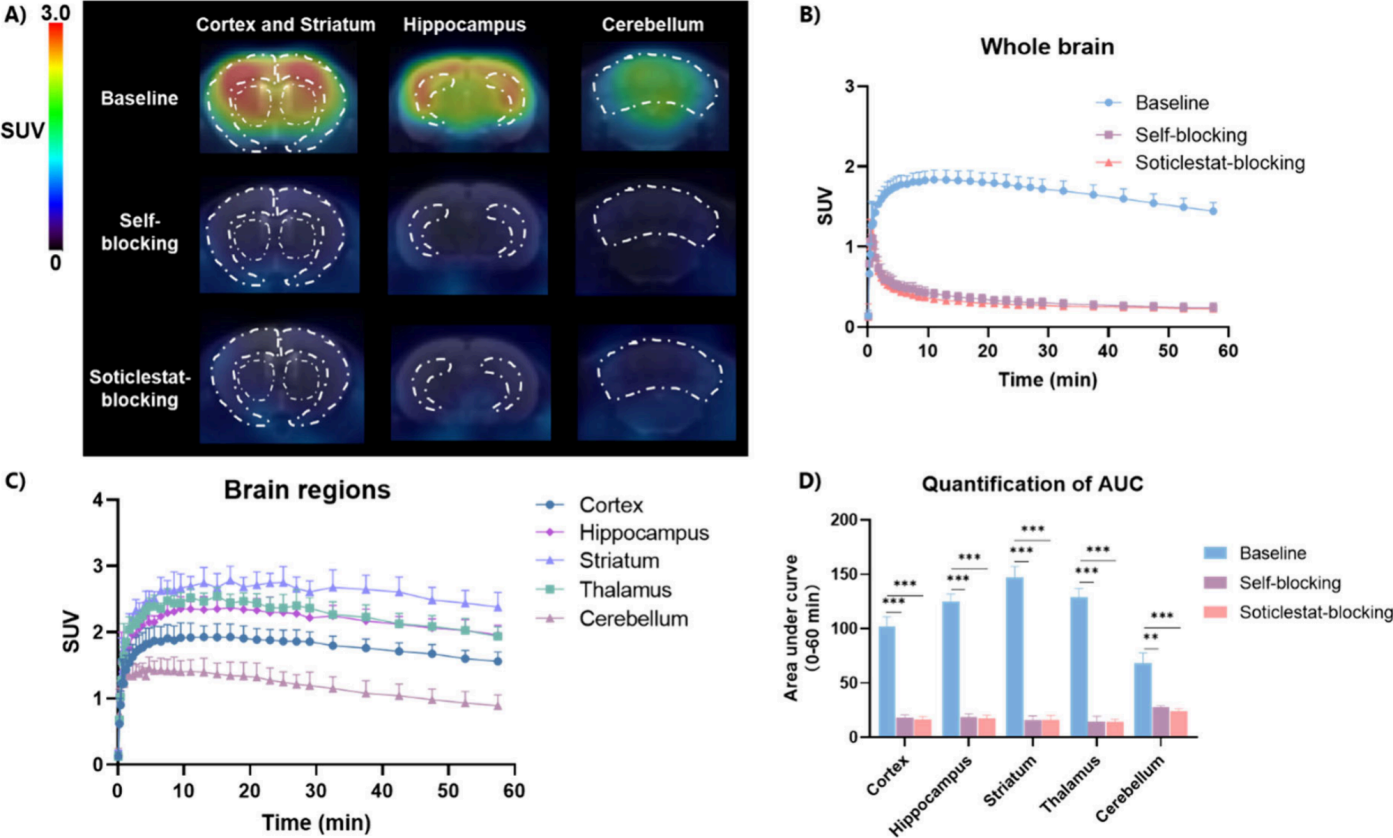
PET imaging studies in the mouse brain. (A) Summed PET images (0–60 min) and (B) whole-brain TACs of [^18^F]**5** in mice under baseline and blocking conditions. (C) Regional TACs of selected brain regions under baseline conditions. (D) Quantification of area under curve (AUC) values for baseline and blocking conditions in mice. Asterisks (*) indicate statistical significance (**p ≤ 0.01, ***p ≤ 0.001). Values represent mean ± SD, *n* ≥ 2.

### Whole-Body Biodistribution Study

To assess the biodistribution profile of [^18^F]**5**, *ex vivo* whole-body biodistribution studies were performed in mice at four time points (5, 15, 30, and 60 min). As shown in Figure 6, [^18^F]**5** demonstrated high initial brain uptake at 5 min (8.7% ID/g), followed by a slight decline yet remaining above 7% ID/g up to 60 min, consistent with the PET imaging results. High uptake was also detected in the small intestine, kidneys, and liver, suggesting that the tracer is primarily cleared through hepatobiliary and urinary pathways. Importantly, bone radioactivity remained low (<1% ID/g throughout 60 min), reflecting minimal *in vivo* defluorination.

**Figure 6:**
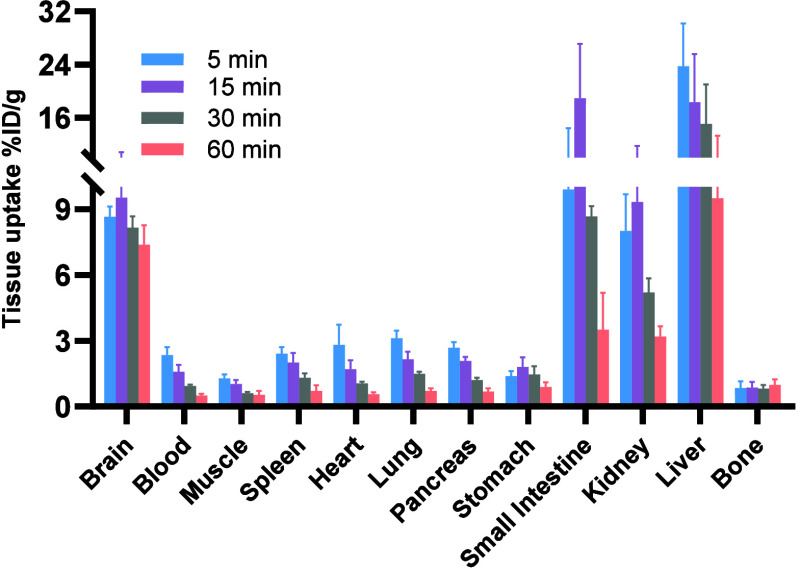
Whole-body *ex vivo* biodistribution of [^18^F]**5** was measured in mice at 5, 15, 30, and 60 min postinjection. Results are given as %ID/g (mean ± SD, *n* = 4), indicating the percentage of the injected dose contained per gram of wet tissue.

## Conclusions

CYP46A1 PET tracers represent a highly promising tool for assessing brain cholesterol homeostasis, a process closely linked to multiple CNS disorders. In this work, we developed and evaluated the deuterated CYP46A1 radioligand [^18^F]**5** (also known as [^18^F]­CHL-2205*-d*_3_) as an *N-*[methyl-*d*_3_] analog of the clinically validated tracer [^18^F]­CHL-2205. The design was motivated by the prospect of attenuating oxidative *N-*demethylation at the amide nitrogen through a deuterium kinetic isotope effect, thereby further stabilizing the scaffold without altering target engagement. Compound **5** exhibited subnanomolar inhibition of CYP46A1, favorable CNS drug-like and ADME properties, and negligible off-target binding. [^18^F]**5** was prepared in practical radiochemical yields and high molar activities and showed robust brain time-activity curves in mice, with a regional distribution that closely matches known CYP46A1 expression patterns. Radiometabolite analysis demonstrates that brain radioactivity is primarily derived from intact parent tracer, consistent with excellent CNS metabolic stability. When compared with [^18^F]­CHL-2205, [^18^F]**5** preserved rather than fundamentally altered the imaging characteristics of this chemotype, suggesting that *N-*demethylation was not a major determinant of tracer clearance *in vivo*. Collectively, these findings establish [^18^F]**5** as a valuable addition to the CYP46A1 PET toolkit and provide mechanistic insight into the metabolic behavior of the CHL-2205 scaffold.

## Experimental Section

### Materials and Methods

All reagents and solvents were obtained from commercial suppliers and used without further purification. Compound **5** and its labeling precursor **13** were synthesized according to our previously reported procedures, with full experimental details provided in the Supporting Information.^32^ All animal studies were reviewed and approved by the Emory University Institutional Animal Care and Use Committee (IACUC; protocol numbers PROTO202200003 and PROTO202200076) and performed in strict compliance with institutional ethical standards. Female CD-1 mice (22–24 g; 5–6 weeks old; strain code 022; Charles River Laboratories) were housed in a temperature- and humidity-controlled facility on a 12-h light/dark cycle with free access to food and water.

### Radiochemistry

[^18^F]­Fluoride was produced via the ^18^O­(*p,n*)^18^F reaction on a GE PETrace 880 cyclotron (GE Healthcare) using 18 MeV protons and >98% enriched [^18^O]­H_2_O. On average, approximately 30 mCi of [^18^F]­Fluoride was trapped from the H_2_^18^O target water on a Sep-Pak QMA Plus Light cartridge (Waters, cat. no. 186004540) and subsequently eluted in the reverse direction using a solution of Et_4_NHCO_3_ (TEAB, 1 mg) in MeOH (1.0 mL). The resulting [^18^F]­fluoride solution was azeotropically dried at 110 °C under a nitrogen stream with the addition of anhydrous MeCN (1.0 mL). A solution containing boronic ester precursor **13** (2 mg) and [Cu­(Py)_4_OTf_2_] (8 mg) in dry DMAC/nBuOH (200/100 μL) was then added, and the reaction mixture was heated at 110 °C for 15 min with the reaction vial uncapped. The crude mixture was diluted with HPLC mobile phase and purified by semipreparative HPLC using a Phenomenex Luna C18(2) column (5 μm, 10 × 250 mm) with elution by MeCN/H_2_O (35/65, v/v, containing 0.1% NEt_3_). The total synthesis time was 90 min (including HPLC purification), affording a nondecay-corrected radiochemical yield of 31.5 ± 1.5% at the start of synthesis (SOS). Radiochemical purity, chemical identity, and molar activity of [^18^F]**5** were assessed using an analytical radio-HPLC system (Agilent 1100 series) equipped with a Waters XBridge C18 column (5 μm, 4.6 × 150 mm), a UV detector set at 254 nm, and a LabLogic in-line radioactive detector. Elution was performed using a MeCN/H_2_O gradient (40/60, v/v, containing 0.1% NEt_3_ trifluoroacetic acid), and the retention time of [^18^F]**5** was approximately 9.5 min.

### *In Vitro* Autoradiography

*In vitro* autoradiography was performed on 20 μm cryosectioned rat brain tissues embedded in Tissue-Tek O.C.T. and stored at −80 °C until use. In brief, brain sections were pre-equilibrated in 200 mL of buffer 1 (50 mM Tris, 0.1% BSA) for 10 min at room temperature. Sections were then incubated for 30 min in buffer 1 containing [^18^F]**5** (1 μci/mL). For blocking studies, sections were coincubated with 10 μM unlabeled compound **5** or soticlestat. Following incubation, sections were washed sequentially in buffer 1 (1 × 5 min) and buffer 2 (50 mM Tris without BSA; 2 × 2 min). Sections were then briefly dipped twice (5 s each) in distilled water, air-dried, and exposed to a phosphor imaging plate (BAS-MS2025, GE Healthcare) for 12 h. The plates were scanned using an Amersham Typhoon system (Cytiva, USA).

### PET Imaging

PET studies were performed in anesthetized CD-1 mice using a Genisys G8 PET scanner (Sofie Biosciences, USA). Following intravenous tail-vein administration of [^18^F]**5** (1.5–3.0 MBq), dynamic PET images were acquired for 60 min. Image reconstruction and quantitative analysis were conducted using PMOD software (version 4.3; PMOD Technologies, Switzerland). For blocking experiments, compound **5** or Soticlestat was administered 10 min prior to [^18^F]**5** injection.

### Whole-Body Biodistribution

In brief, CD-1 mice (n = 4) were administrated with an intravenous tail-vein injection of [^18^F]**5** (0.5 MBq in 0.1 mL). At 5, 15, 30, and 60 min postinjection, the animals were euthanized by cervical dislocation, and the selected tissues were harvested and weighed. Decay-corrected radioactivity in each organ was quantified using a Wizard automatic gamma counter (PerkinElmer, USA).

### Radiometabolite Analysis

CD-1 mice were intravenously injected with [^18^F]**5** (15 MBq, 0.1 mL) and euthanized by decapitation at 30 min postinjection (n = 2). Brain tissue and plasma were rapidly collected, quenched with acetonitrile, and centrifuged. A KNAUER semipreparative HPLC system was used to collect the fractions, and the radioactivity of each fraction was quantified using a Wizard automatic gamma counter. Data were subsequently analyzed and plotted using GraphPad. The percentage of unchanged [^18^F]**5** was calculated from the chromatograms as % parent = (peak area of [^18^F]**5**/total radioactivity peak area) × 100.

### Safety

No unexpected or unusually high safety hazards were encountered.

## Supplementary Material



## Notes

Experimental details for the synthesis of compounds **5** and **13** (Scheme S1–S8); Off-target binding assay of compound **5** (Figure S1) (PDF)

## Abbreviations

BBB: blood–brain barrier
CNS: central nervous system
CYP11A1: cholesterol side-chain cleavage enzyme
CYP27A1: sterol 27-hydroxylase
CYP7A1: cholesterol 7α-hydroxylase
CYP46A1 or CH24H: cholesterol 24-hydroxylase
24-OHC: 24-hydroxycholesterol
AD: Alzheimer’s disease
LXR: liver X receptor
Aβ: amyloid-β
HD: Huntington’s disease
PET: positron emission tomography
NHPs: nonhuman Primates
ADME: Absorption, Distribution, Metabolism, Excretion
DMI: deuterium metabolic imaging
MW: molecular weight
TPSA: Topological Polar Surface Area
HBD: hydrogen-bond donors
MPO: multiparameter optimization
MDCK: Madin–Darby canine kidney
MDR1: human multidrug resistance 1
P_app_: apparent permeability coefficient
DMAC: *N,N*-dimethylacetamide
TACs time: activity curves
AUC: area under the curve
%ID/g: percent injected dose per gram of tissue
SD: Standard Deviation
MR: Magnetic Resonance
QMA: Quaternary Methyl Ammonium
HPLC: High-Performance Liquid Chromatography
SOS: start of synthesis.
