# Supplementary material for: Radiosynthesis and Evaluation of 18F‑Labeled Deuterated Radioligand for Positron Emission Tomography Imaging of Cholesterol 24-Hydroxylase
Source: ACS Med Chem Lett. 2026 Jan 20;17(2):538–46. doi: 10.1021/acsmedchemlett.5c00740 (PMC12907933; doi:10.1021/acsmedchemlett.5c00740)
Supplement: Supplementary file 1 [file ml5c00740_si_001.pdf]

Supporting Information

Radiosynthesis and Evaluation of ^{18}F-Labeled Deuterated Radioligand for Positron Emission Tomography Imaging of Cholesterol 24-Hydroxylase

Yinlong Li,[1,†,*] Zhendong Song,[1,†] Haofeng Shi,[1] Taoqian Zhao,[1] Jiahui Chen,[1] Xin Zhou,[1] Qilong Hu,[1] Xiaoyan Li,[1] Lingxin Meng,[1] Ruihu Song,[1] Zhenkun Sun,[2] Chongjiao Li,[1] Achi Haider,[1] Hongjie Yuan,[2] Steven H. Liang[1,3*]

[1]Department of Radiology and Imaging Sciences, Emory University, 1364 Clifton Road, Atlanta, Georgia 30322, United States

[2]Department of Pharmacology and Chemical Biology, Emory University School of Medicine, Atlanta, Georgia, 30322, United States

[3]Wallace H. Coulter Department of Biomedical Engineering, Georgia Institute of Technology and Emory University, Atlanta, GA 30332, United States

[†]These authors contributed equally

Corresponding Authors

Yinlong Li − *Department of Radiology and Imaging Sciences, Emory University, 1364 Clifton Road, Atlanta, Georgia 30322, United States.* Email: yinlong.li@emory.edu.

Steven H. Liang − *Department of Radiology and Imaging Sciences, Emory University, 1364 Clifton Road, Atlanta, Georgia 30322, United States.* Email: steven.liang@emory.edu.

Content

1) Synthesis of compounds **5** and **13**.
2) Off-target binding assay of compound **5**.

General Information

Nuclear magnetic resonance (NMR) spectra were acquired on a Bruker AVANCE NEO 400 MHz spectrometer operating at 400 MHz for ^{1}H and 100 MHz for ^{13}C. Chemical shifts (δ) are reported in parts per million (ppm) and referenced to tetramethylsilane (TMS) using residual solvent peaks. Peak multiplicities are designated as follows: singlet (s), doublet (d), triplet (t), quartet (q), and multiplet (m). HPLC–MS analyses were performed on an Agilent 6120B single-quadrupole mass spectrometer equipped with an electrospray ionization (ESI) source. Analytical HPLC was conducted on an Agilent 1100 system with a G1315A diode-array detector. High-resolution mass spectrometry (HRMS) was carried out using a Thermo Fisher Scientific UPLC-ESI-Q-Orbitrap mass spectrometer.

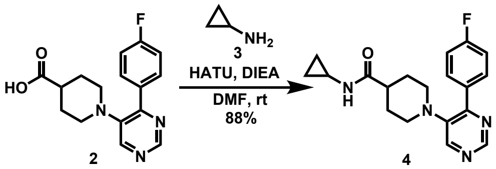

Scheme S1. Synthesis of compound **4**.

*Synthesis of N-cyclopropyl-1-(4-(4-fluorophenyl)pyrimidin-5-yl)piperidine-4-carboxamide (**4**)*

To a stirred mixture of 1-(4-(4-fluorophenyl)pyrimidin-5-yl)piperidine-4-carboxylic acid **2** (50 mg, 0.16 mmol) and cyclopropanamine **3** (10.42 mg, 0.18 mmol) in DMF (1 mL) was added HATU (77.57 mg, 0.2 mmol) and DIEA (53.61 mg, 0.41 mmol) at 25 ^{o}C for 2 h. The reaction mixture was poured into water and extracted with EtOAc (10 mL*3), washed with water and brine, dried over Na_2SO_4, concentrated and purified by flash (DCM/MeOH=95/5) to give N-cyclopropyl-1-(4-(4-fluorophenyl)pyrimidin-5-yl)piperidine-4-carboxamide **4** (50 mg, 88%) as a yellow solid. LCMS (ESI): m/z, 341.1 $[M+H]^+$.

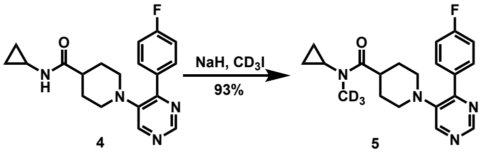

Scheme S2. Synthesis of compound **5**.

*Synthesis of N-cyclopropyl-1-(4-(4-fluorophenyl)pyrimidin-5-yl)-N-(methyl-d3)piperidine-4-carboxamide (**5**)*

To a stirred mixture of N-cyclopropyl-1-(4-(4-fluorophenyl)pyrimidin-5-yl)piperidine-4-carboxamide **4** (40 mg, 0.12 mmol) in THF (1 mL) was added 60% NaH (14.4 mg, 0.59 mmol) at 0 ^{o}C for 1 h. To above mixture was added CD_3I (2.17 mg, 0.015 mmol) before stirring at rt for 18

h. The reaction solution was poured into water, and extracted with EtOAc (10 mL*3), washed with water and brine, dried over Na_2SO_4, concentrated and purified by flash (DCM/MeOH=98/2~95/5) to give N-cyclopropyl-1-(4-(4-fluorophenyl)pyrimidin-5-yl)-N-(methyl-d3)piperidine-4-carboxamide **5** (40 mg, 93%) as an off white solid. ^{1}H NMR (400 MHz, Chloroform-*d*) δ 8.91 (s, 1H), 8.43 (s, 1H), 8.26 – 8.07 (m, 2H), 7.18 (dd, J = 9.7, 7.6 Hz, 2H), 3.31 (d, J = 11.6 Hz, 2H), 3.11 (t, J = 11.7 Hz, 1H), 2.76 – 2.62 (m, 3H), 1.89 (dt, J = 13.2, 6.5 Hz, 2H), 1.72 (d, J = 13.1 Hz, 2H), 0.90 (*t*, J = 6.2 Hz, 2H), 0.75 (p, J = 5.0 Hz, 2H). ^{13}C NMR (100 MHz, Chloroform-*d*) δ 177.40, 164.90, 162.41, 156.62, 152.44, 147.00, 144.05, 133.87 (d, J = 3.4 Hz), 130.30 (d, J = 8.4 Hz), 115.65 (d, J = 21.5 Hz), 50.59, 38.83, 30.89, 28.41, 9.02. LCMS: 358.2 $[M+H]^+$. HRMS (ESI): exact mass calcd for $C_{20}H_{21}D_3FN_4O^+$ $[M+H^+]$, 358.2117; found, 358.2109.

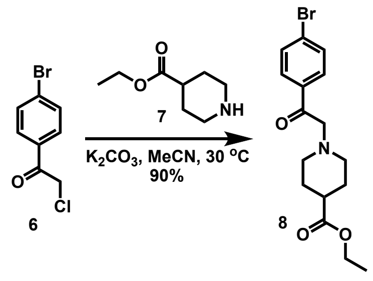

Scheme S3. Synthesis of compound **8**.

Synthesis of ethyl 1-(2-(4-bromophenyl)-2-oxoethyl)piperidine-4-carboxylate (**8**).

To a stirred mixture of 1-(4-bromophenyl)-2-chloroethan-1-one **6** (20.0 g, 85.66 mmol) and ethyl piperidine-4-carboxylate **7** (14.14 g, 89.94 mmol) in MeCN (200 mL) was added K_2CO_3 (17.76 g, 0.13 mol) at 25 °C overnight. The reaction mixture was concentrated and purified by flash (PE/EtOAc = 4/1) to give ethyl 1-(2-(4-bromophenyl)-2-oxoethyl)piperidine-4-carboxylate **8** (27.3 g, 90%) as a yellow solid and used directly for the next step.

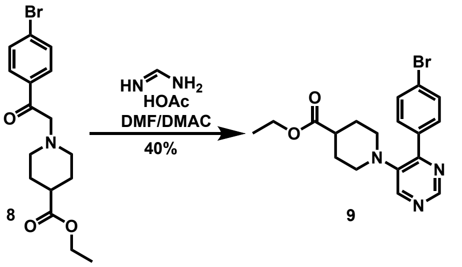

Scheme S4. Synthesis of compound **9**.

Synthesis of ethyl 1-(4-(4-bromophenyl)pyrimidin-5-yl)piperidine-4-carboxylate (**9**).

To a solution of ethyl 1-(2-(4-bromophenyl)-2-oxoethyl)piperidine-4-carboxylate **8** (24.0 g, 67.75 mmol) and formamidine acetate (10.45 g, 0.23 mol) in DMF/DMAC (20 mL) before being heated to 109 °C and stirred for 18 h. The reaction mixture was cooled to rt and evaporated. DIEA (80 mL) and *n*-BuOH (80 mL) were added before heated to 100 °C for 18 h. The mixture was cooled to rt, poured into ice water and extracted with EtOAc (10 mL*3). The organic layer was washed with brine, dried over Na_2SO_4, concentrated and purified by flash (PE/EtOAc/DCM = 6/1/1) to give ethyl 1-(4-(4-bromophenyl)pyrimidin-5-yl)piperidine-4-carboxylate **9** (12 g, 40%) as a light yellow solid and used directly for the next step.

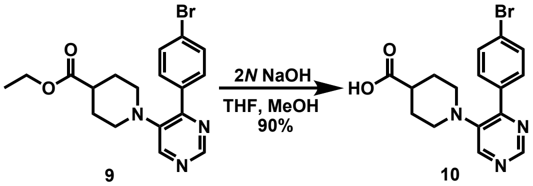

Scheme S5. Synthesis of compound **10**.

Synthesis of 1-(4-(4-bromophenyl)pyrimidin-5-yl)piperidine-4-carboxylic acid (**10**).

To a solution of ethyl 1-(4-(4-bromophenyl)pyrimidin-5-yl)piperidine-4-carboxylate **9** (10 g, 25.62 mmol) in MeOH/THF (4 mL/4 mL) was added NaOH (1.02 g, 25.62 mmol) before stirred at rt for 3 h. The solution was concentrated and acidified to pH = 2~3 and filtered to give 1-(4-(4-bromophenyl)pyrimidin-5-yl)piperidine-4-carboxylic acid **10** (8 g, 90%) as a yellow solid.

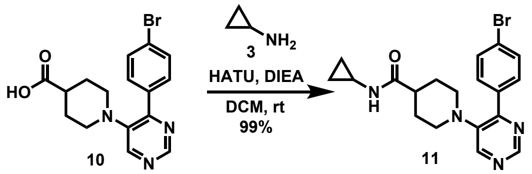

Scheme S6. Synthesis of compound **11**.

Synthesis of 1-(4-(4-bromophenyl)pyrimidin-5-yl)-N-cyclopropylpiperidine-4-carboxamide (**11**).

To a stirred mixture of 1-(4-(4-bromophenyl)pyrimidin-5-yl)piperidine-4-carboxylic acid **10** (500 mg, 1.38 mmol) and cyclopropanamine **3** (157.61 mg, 2.76 mmol) in DCM (4 mL) was added HATU (1.05 g, 2.76 mmol) and DIEA (553.2 mg, 4.14 mmol) at 25 °C for 5 h. The reaction mixture was poured into water and extracted with EtOAc (10 mL*3), washed with water and brine, dried over Na_2SO_4, concentrated and purified by flash (PE/EtOAc = 1/1) to give 1-(4-(4-

bromophenyl)pyrimidin-5-yl)-N-cyclopropylpiperidine-4-carboxamide **11** (550 mg, 99%) as a yellow solid.

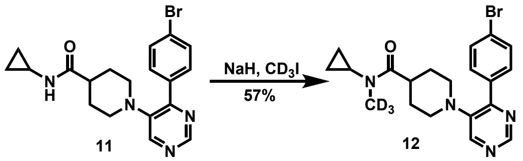

Scheme S7. Synthesis of compound **12**.

Synthesis of 1-(4-(4-bromophenyl)pyrimidin-5-yl)-N-cyclopropyl-N-(methyl-d3)piperidine-4-carboxamide (**12**).

To a stirred mixture of 1-(4-(4-bromophenyl)pyrimidin-5-yl)-N-cyclopropylpiperidine-4-carboxamide **11** (500 mg, 1.12 mmol) in THF (7 mL) was added 60% NaH (133.82 mg, 5.58 mmol) at 0 °C for 1 h. To the above mixture was added CD_3I (474.8 mg, 3.35 mmol) before stirred at rt for 18 h. The reaction solution was poured into water, and extracted with EtOAc (10 mL*3), washed with water and brine, dried over Na_2SO_4, concentrated and purified by flash (PE/EtOAc = 1/1) to give 1-(4-(4-bromophenyl)pyrimidin-5-yl)-N-cyclopropyl-N-(methyl-d3)piperidine-4-carboxamide **12** (300 mg, 57%) as a yellow solid.

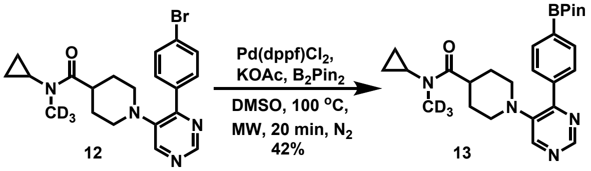

Scheme S8. Synthesis of compound **13**.

Synthesis of N-cyclopropyl-N-(methyl-d3)-1-(4-(4-(4,4,5,5-tetramethyl-1,3,2-dioxaborolan-2-yl)phenyl)pyrimidin-5-yl)piperidine-4-carboxamide (**13**).

To a stirred mixture of 1-(4-(4-bromophenyl)pyrimidin-5-yl)-N-cyclopropyl-N-(methyl-d3)piperidine-4-carboxamide **12** (150 mg, 0.36 mmol), B_2Pin_2 (136.58 mg, 0.54 mmol) and KOAc (105.56 mg, 1.08 mmol) in DMSO (2 mL) was added $Pd(dppf)Cl_2$ (26.31 mg, 0.04 mmol) before heat to 100 °C and microwave for 20 minutes under N_2. The reaction solution was poured into water, and extracted with EtOAc (20 mL*3). The organic phase was washed three times with water, dried over Na_2SO_4, concentrated and purified by flash (PE/EtOAc = 1/1) to give N-cyclopropyl-N-(methyl-d3)-1-(4-(4-(4,4,5,5-tetramethyl-1,3,2-dioxaborolan-2-yl)phenyl)pyrimidin-5-yl)piperidine-4-carboxamide **13** (70 mg, 42%) as a light yellow solid. ^{1}H NMR (400 MHz,

Chloroform-*d*) δ 8.91 (s, 1H), 8.41 (s, 1H), 8.08 (d, J = 8.0 Hz, 2H), 7.96 – 7.85 (m, 2H), 3.30 (d, J = 11.6 Hz, 2H), 3.07 (t, J = 11.7 Hz, 1H), 2.75 – 2.55 (m, 3H), 1.97 – 1.78 (m, 2H), 1.73 – 1.61 (m, 2H), 1.36 (s, 12H), 0.88 (dd, J = 7.1, 2.1 Hz, 2H), 0.80 – 0.66 (m, 2H). ^{13}C NMR (100 MHz, Chloroform-*d*) δ 177.45, 157.56, 152.11, 146.55, 144.26, 140.38, 134.97, 127.18, 83.97, 50.53, 38.84, 30.88, 28.36, 24.93, 9.00. LCMS: 466.3 [M+H]$^+$. HRMS (ESI): exact mass calcd for $C_{26}H_{33}D_3BN_4O^{3+}$ [M+H$^+$], 466.3063; found, 466.3062.

(A)

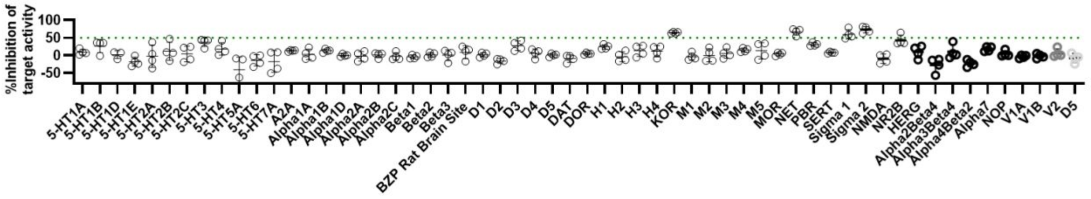

(B)

Target	Seconndary assay Ki (nM)
**KOR**	2979.20
**NET**	> 10,000
**Sigma 1**	4319.17
**Sigma 2**	3232.21

Figure S1. (A) Off-target pharmacological evaluation of **5** at a concentration of 10 μM against major CNS targets, including common GPCRs, enzymes, ion channels and transporters. All data are mean ± SD, n = 4. (**B**) Secondary radioligand binding assays for the targets with >50% of inhibition on primary screening.
